# The Role of Food in the Family Relationships of Adolescents With Anorexia Nervosa and Bulimia in Northeastern Brazil: A Qualitative Study Using Photo Elicitation

**DOI:** 10.3389/fpsyt.2021.623136

**Published:** 2021-04-16

**Authors:** Juniana de Almeida Mota Ramalho, Mayssa' El Husseini, Lucas Bloc, Julia Sursis Nobre Ferro Bucher-Maluschke, Marie Rose Moro, Jonathan Lachal

**Affiliations:** ^1^Université of Paris 13, Université Sorbonne Paris Cité, URTPP - Unité transversale de psychogénèse et psychopathologie, Villetaneuse, France; ^2^University Grenoble Alpes, LIP/PC2S, Grenoble, France; ^3^University of Picardie Jules Verne, CHSSC, Amiens, France; ^4^APHP, Hôpital Cochin, Maison de Solenn, Paris, France; ^5^University of Fortaleza (UNIFOR), APHETO - Laboratório de Psicopatologia e Clínica Humanista-Fenomenológica, Fortaleza, Brazil; ^6^Centro de Ensino Unificado de Brasília - CEUB, Brasilia, Brazil; ^7^Université de Paris, PCPP, Boulogne-Billancourt, France; ^8^Université Paris-Saclay, UVSQ, Inserm, CESP, Team DevPsy, Villejuif, France; ^9^Service de Psychiatrie de l'Enfant et de l'Adolescent, CHU de Clermont-Ferrand, Clermont-Ferrand, France; ^10^Université Clermont Auvergne, Clermont-Ferrand, France

**Keywords:** adolescent, food, family, anorexia nervosa, bulimia nervosa, photo elicitation

## Abstract

Family components can play roles both as protective factors and maintenance mechanisms of eating disorders. We aimed to investigate the role of food in the family relationships of adolescents with anorexia nervosa and bulimia in northeastern Brazil. Using photo elicitation, a visual narrative method that gives insight into the participants' perspective through photograph, we conducted semi-structured interviews with 26 people: four teenage girls with anorexia, four with bulimia, eight mothers, four fathers, five grandmothers, and one sister. Data were analyzed using the principles of Interpretative Phenomenological Analysis, which highlighted the following themes: control of the parent-adolescent relationship through food; food as a mean of experiencing parental presence-absence; food as the focus of conflict in the nuclear family, and food as a source of three-generational conflict. Food seemed to be a means for teens and parents to express physical suffering and psychological violence. Moreover, mourning appeared to influence the girls' relationships with food. Conflict in these families is not focused solely on food, but extends to other subjects, and teenagers' emotional reactivity concerning their relationship with their parents and food during family mealtimes varied. These features reinforced the cultural aspect and influences of eating experiences among adolescent girls with eating disorders. Remarkable disparities exist in the generations' views on what rules and rituals these adolescents must follow at meals. These disparities can obfuscate generational boundaries in these families. Our data reinforce the need to focus on the adolescent's autonomy in the family setting and on family identity as related to food among three generations. These findings necessitate a reorganization of boundaries between these generations.

## Introduction

Eating disorders are psychiatric conditions associated with individual, family, and sociocultural factors (1, 2). They are also associated with lower quality of life and increased health care use (3, 4). There is a 12-month prevalence of bulimia nervosa (BN) of 1%−1.5% among adolescent girls (5). A systematic review has indicated weighted population means and ranges of lifetime prevalence of anorexia nervosa (AN) as 1.4% (0.1–3.6%) for women, including adolescents (6). Studies from Argentina, Brazil, Chile, Colombia, Mexico, and Venezuela report a mean point-prevalence rate of 0.1% for anorexia nervosa and 1.16% for bulimia nervosa in the general population (7). Specifically, in northeastern Brazil, bulimic and anorexic symptoms have increased dramatically in recent years in diverse socioeconomic contexts (8). Surveys of school aged children (10–14 years old) have reported that 1.3% are dissatisfied with their body image and practice restrictive eating, while 0.6% present both compulsive and restrictive eating as well as dissatisfaction with their body image (8).

Previous data suggest that family components can play an important role both in the complex pathogenic and maintenance mechanisms of eating disorders (9, 10). Other studies have described family models in the context of AN and BN. For instance, a “model of psychosomatic family” was conceptualized for families with a member presenting anorexia nervosa. According to this model, these families are enmeshed, rigid and conflict avoidant (11). This model is based on the structural family therapy founded by Minuchin (12), appointing the development of boundaries as an important parameter for evaluation of family structures.

Literature about family relationships of BN patients shows that the connection between mothers and adolescents was negatively associated with BN symptoms (13). Previous studies have described that family environment of BN patients is marked often by disengagement, in which members are conflicted, and lack of emotional expression (14, 15). Other studies do not underpin that all families with a child diagnosed with any eating disorder have higher levels of enmeshment and conflict than those without (16, 17). The Academy of Eating Disorders has declared against any etiological model of eating disorders influencing the idea that family could be the primary cause of AN or BN (18).

Several studies have examined the associations between family meal patterns and disordered eating in the general population (19, 20). Patients with AN report higher rates of total family meal frequency as compared with patients with BN. It shows that parental vigilance over the eating of patients with AN may be relatively more frequent whereas families of BN patients may be less aware of their eating disorder behaviors and, consequently, watch less their children during family meals (21). Based on these assumptions, numerous studies have highlighted the importance of parental involvement in adolescent treatment (22, 23).

Studies have shown that adolescents with eating disorders and their parents manifest dissatisfaction about their family relationships (24, 25). Nevertheless, understanding the role food occupies in the family relationships with teenage girls is still little explored. Furthermore, these psychopathological mechanisms are complex and differ according to cultural contexts (26). A better comprehension about the experience with food in family mealtimes is necessary to elaborate more adequate therapeutic strategies in the treatment. This issue was the starting point of a research project conducted by our team: to study the interrelations between eating disorders and family relationships in adolescence in different cultural contexts. Several studies have been carried out in normal and pathological contexts both in France and Brazil (27–31), using photo elicitation, a visual narrative method which consists of using one or several photographs taken by the subject as the basis of the interview (32).

In this study, we propose to investigate the role of food in the family relationships of adolescents with anorexia nervosa and bulimia in northeastern Brazil through photo elicitation, what the issues are, and what adaptations can be suggested.

## Materials and Methods

Our observational and exploratory study is a qualitative study, a type of research appropriate for exploring complex phenomena within contexts of subjects (33). It has shown its value in psychology (34, 35) and in the field of eating disorders (36, 37).

### Participants

This research included a convenience sample of eight girls aged 12–18 years with anorexia nervosa or bulimia nervosa. We interviewed these adolescents, their parents (*N* = 12), and, unlike in our previous work (27–30), their grandmothers (*N* = 05) and one sister. This decision was based on specific aspects of Brazilian culture: the closeness of extended and nuclear families, linked by concepts of loyalty and responsibilities (38). Accordingly, the personal choices of nuclear families may be influenced by their extended families.

We recruited the participants in two public medical centers in Fortaleza, capital of the State of Ceará, Brazil: *Centro de Estudos e Tratamento de Transtornos Alimentares* (CETRATA) and *Programa de Nutrição e Tratamento aos Transtornos Alimentares* (PRONUTRA). The recruited adolescent girls were in treatment at these two medical centers (Table 1).

**Table 1:** Participants' characteristics.

**Subject**	**Birth city**	**Age**	**ED**	**BMI**	**Parental situation**	**Sibling**	**Interviewed**	**Context of the picture**
Flora	Fortaleza	12	AN	11.9	Married	1 S	F, M	Eating with parents and sister
Valentina	Acaraju	17	AN	13.4	Married	1 S	F, M, GM	Eating with parents and sister
Clara	Belém	17	AN	15.2	Divorced	1 S, 4 B	M	Eating alone
Gisele	Fortaleza	17	AN	18.1	Divorced	1 B	F, M, GM	Eating with grandmother, grandmother's husband and her brother
Daniela	Recife	16	BN	22.2	Divorced	0	M, GM	Eating with mother
Ester	Maracanaú	17	BN	30.5	Married	0	M, GM	Eating with mother
Alice	Fortaleza	15	BN	25.1	Divorced	2 S	F, M, GM, S	Eating alone
Dora	Maranguape	16	BN	23.8	Divorced	1 B	M	Eating alone

*ED, eating disorder; BMI, body mass index; AN, anorexia nervosa; BN, bulimia nervosa; F, father; M, mother; GM, grandmother; S, sister; B, brother*.

### Data Collection

The data presented here were collected through semi-structured interviews facilitated by photo elicitation. Social and demographic information came from a preliminary recruitment interview. At that time, we also gave the girls the following instructions:

“*We want you to take a photograph of the table after a family meal. It should be taken before the table is cleared. There should not be anyone visible in the photograph, which means you should take it after the people at the meal have left the table. You can take as many pictures as you like, but in the end, you will choose just one to comment on with the researcher in the interview.”*

The photograph chosen by the teen was used as the basis for all interviews. Images enable participants to elicit deeper elements of human consciousness than words do (32). Moreover, the subjects participate in the production of the photograph, which amplifies their subjective connection with the image (39). We interviewed the adolescent first, then each relative separately. As required by the ethics committee, the photographs were neither published nor stored.

The interviews were semi-structured, with an open-ended approach (Table 2). The interviews started with a description of and comment on the meal represented in the photograph, followed by a discussion of how meals usually unfolded and what role food had in family relationships. Each interview lasted from one to one and a half hours. The interview sheets were anonymized, and pseudonyms have been used to preserve participants' privacy.

**Table 2:** Guide for semi-structured interviews.

**Schedule interview**
1. Description of the picture of family mealtime
-*Would you describe the meal in detail, and what happens during a meal?*
-*How are meals organized at home?*
2. Description of feelings during family mealtime
-*Would you tell me about how you felt, your emotions, before, during and after the family meal you photographed?*
3. Food and memory
-*Would you tell me about two family meals you particularly remember?*
-*How did you feel in these family meals?*
4. Food and transmission
-*How is it important to transmit recipes in your family?*

### Data Analysis

All interviews were audio recorded, transcribed verbatim in Portuguese, and analyzed according to the framework of Interpretative Phenomenological Analysis (IPA) (40, 41).

The aim of IPA is to explore in depth how participants make sense of their personal and social world by using a process that combines descriptive and interpretative components (41). It is based on the concept of double hermeneutics: the participants try to make sense of the experience, upon which the researcher makes his/her own interpretation (41, 42).

The data analysis consisted of six steps. In the first step, we read and re-read each transcript. In the second step, we realized descriptive comments, in which the content of what each participant had said was described; as well as linguistic comments, focusing on the words and expressions used; and conceptual comments engaging at a more conceptual level (41).

The third step involved identifying emergent themes, formed centrally from the conceptual comments, and condensing them in order to reflect both the participants' words and the analysts' interpretation. In the fourth step, we established connections across emergent themes for each case. There was a dynamic and interactive process to apprehend ways in which participants' narratives were similar but also different. We identified patterns and grouped them under superordinate themes. The fifth step involved repeating the four steps described for each case. The final stage entailed looking for patterns across cases. We fixed superordinate themes shared across all interviews (41).

The analysis was performed in Portuguese by a single researcher (JR) and the direct quotations were translated into English only for this paper. The transcripts were read by two other researchers (JL and MEH) to check consistency and coherence. The data were organized using NVivo 11 software for the data analysis.

### Ethical Approval and Consent to Participate

The study was approved under number 607.183 by the Ethics Review Committee of *Universidade Federal do Ceará* in Fortaleza, Brazil, at 15th October 2013, according to Resolution 466 of the National Council, and written informed consent was obtained from all participants and parents where necessary (in the case of minors).

## Results

The analysis was organized around two superordinate themes: the role of food in the parent-child relationship and the role of food in the family group dynamics. The first one has two themes: the experience of control in parent-adolescent relationships through food and food as a means of experiencing parental presence-absence. The second one is grouped in two other themes: food as a focus of conflict in the nuclear family and food as a source of three-generational conflicts.

First superordinate theme: The role of food in the relationship parent-child

- The experience of control of the parent-adolescent relationship through food

This theme describes the use of food by both adolescents with anorexia or bulimia and their parents to try to control their relationships. Power struggles and the expression of feelings, such as sadness and anger, marked their experiences with food. Adolescents and parents simultaneously confronted and avoided each other at meals. The conflict perpetuated both the anorexic and bulimic symptoms. The teens with bulimia nervosa had negative feelings related to their parents' ambivalent attitudes—ambivalence that led them to complain both when the girls ate and when they did not. They seemed to have had more difficulties than those with anorexia in expressing their feelings. Daniela, for example, after being asked how she felt about eating, seemed to have a hard time expressing herself while Flora seemed to express in a more directly manner how she felt while eating:

Daniela: *Sometimes, my mother criticizes my eating behavior*. (...) *Sometimes, I feel like eating. She says: don't eat so much...That's why you get fat!* Interviewer: *How do you feel about it?* Daniela: *Even yesterday, it happened. I felt like eating, I got home feeling very hungry. She offered me food but she said it herself: don't eat so much! Do you see it?* (Bulimia nervosa).

Flora: *If I eat alone, I feel good, because nobody watches me, but when I'm with my parents, I don't feel good, because they watch me*. (Anorexia nervosa).

Valentina's mother: *Even if I tell her I'm going to die, she doesn't eat, can you believe it?* [she speaks in an angry tone] (Anorexia nervosa).

Valentina's father: *She was aggressive. She threw food at my feet*! [He raised his voice in a tone of anger] (Anorexia nervosa).

Parental comments about their children's physique during family mealtimes were mentioned as factors which negatively impacted their experiences of eating in the bulimia group:

Ester: *It was boring* [the meal] (...) *Because we ended up arguing*. (...) *He told me that he was ashamed to go out with me when I was fat... too big!* [She feels] *Too bad* (Bulimia nervosa).

The act of eating was associated with memories of physical and psychic violence in anorexia and bulimia. In this example, Clara's mother used to hit her when she was younger:

Clara: *I'm really enraged. Sometimes, I cry with so much rage!* (...) *She lifts her slipper and says: Aren't you eating? So, I start to cry*. (…) *7 years old...8...9, 10, 11, 12, she hit me, because I didn't want to eat. I remember that it was always hard*.... (Anorexia nervosa).

Among adolescents with anorexia, emotions expressed by parents may be a way to promote the act of eating. They may eat to be allowed to experience affection from their parents:

Interviewer: *Tell me about a meal you had with your family that you liked*.

Flora: *It was when my mother was affectionate to me, she hugged me and she thanked me for helping her, because I ate. I felt good*. (Anorexia nervosa).

Clara: *I eat more for my mother*. (...) *I eat because of my mother, because I see her sad, she cried several times, asking me to eat*. (Anorexia nervosa).

Parents described the suffering and concern they experience in monitoring their daughter's eating. Although fathers appeared to exercise greater control at family meals than did their daughters with anorexia nervosa, they described these as moments of torture and discomfort. At the same time, the adolescent described them as “hell.”

Valentina's father: *She says to me: I'll eat by myself*. [He repeats what he said to her]. *It's ok, but my company will be good for you,” because I annoy her, I watch how much she eats …This is at breakfast, at snack time, at lunchtime, and at dinner. (...) For me, it* [the mealtime] *is a moment of torture*. (Anorexia nervosa).

Valentina: *They drive me crazy! They make my life hell!* (Anorexia nervosa).

Flora's father: *That day was too hard, because I insisted to her: I will be here until you eat everything!* (…) *It's too disturbing. It is uncomfortable to stand in front of a person who does not want us to watch her…* (Anorexia nervosa).

Sometimes, parents recalled their own eating experiences with their families when they were younger to decide on the attitude, they should adopt about their daughters' eating behavior. Such experiences influenced how some parents sought to manage their relationships with their daughters. In Valentina's case, her father recalled:

Valentina's father*: I was 16, 17 years old, and I moved to my uncles' home. I was forced to eat things I didn't like*. (…) *Sometimes, I threw the food away and wanted to leave. I was traumatized*. (…) *That's why I work with a variety of food, with things she likes. Why? Because I suffered in my own flesh, my flesh ached from being forced to eat things*

*I didn't like*. (Anorexia nervosa).

- Food as a mean of experiencing parental presence-absence

Eating disorder symptoms were associated with situations of mourning, such as parental divorce or a parent's death, for both patients with anorexia and those with bulimia. Food appeared to compensate for the parents' affective absence. Depression, anxiety, and sleep problems were also described in this context. Clara's parents had separated:

Clara's mother: *My daughter stopped eating. She wanted to kill herself, to die... she was so depressed that she wouldn't get out of bed*. [A long pause] *She loved her father so much*. (Anorexia nervosa).

Ester was talking about the time after her father's death:

Ester: *The amount increased. Before, I ate a little, I ate normally. Now, I wake up at 3 am and I have an urge to eat and I do eat*. (Bulimia nervosa).

Daniela described how her father's presence influenced her mother's attitude toward their meals. Her parents were divorced, and the photo she produced had pans on the table, allowing her to talk about the differences in the food and table arrangements with and without her father.

Daniela: *When he comes to have lunch at my home, it is tidier than this*. *She does not serve the food in the pan but on a plate. When it is only her and me, she makes rice and chicken*. *With my father, she makes many things*. (…) *I don't care so much about it*. (Bulimia nervosa).

Second superordinate theme: The role of food in the family group dynamics

- Food as a source of conflict in the nuclear family

Participants described experiences showing that food did not seem to be a vector of family cohesion. Instead, it seemed to induce conflicts between family members. Parents expressed sadness for this lack of identity through food, including differences in food preferences in the group of anorexia.

Valentina's father: *Lunch is set differently for the three of us. One eats one way, another one eats another way. There is no single dish that everybody accepts. In our home, it is a problem*. (…) *It already shows a conflict*... (Anorexia nervosa).

Clara's mother: *I am too nervous!* (…) *It is a sad mealtime*. [Silence] *We eat with pleasure, she doesn't. (…) I see my daughter forced to eat. She puts something in her mouth that she doesn't want. She isn't happy eating*. (Anorexia nervosa).

Fear is also mentioned as a feeling experienced by family members in the bulimia nervosa group.

Paula (Alice's sister): *When the whole family is eating together, we don't talk about anything, because we are afraid* [discrete laughter]... *So, we prefer to be silent during the meal, we see her eating a lot and you can't say anything, because if you do, she leaves, she doesn't say where she is going*. (Bulimia nervosa).

Food appeared as a source of conflict within the three sub-systems: parents-adolescent, the couple, and the siblings. In the following example, Valentina's mother repeated what she had told her husband when she saw him coming with Valentina carrying a readymade meal. Alice's sister told that her family avoids confrontation with the teenager and expresses feelings of discomfort about not knowing how to help her sister.

Valentina's Mother: *I can't believe what I see! You knew I had prepared Valentina's dinner. You said I was lazy but look, I made it, I cooked, I have prepared a delicious soup for dinner!* (Anorexia nervosa).

Paula (Alice's sister): *We argue a lot, because I don't understand her. I often get the impression that she is lying, that she is inventing things to... to... get the family's attention*. [She feels] *Bad, huh? She's my sister... I wanted to help her* [crying]. *I don't know what to do* [stops crying], *I don't know if she's exaggerating. She doesn't talk*. (Bulimia nervosa).

- Food as a source of three generational conflicts

This analysis explored generational aspects observed during interviews, including grandmothers' interviews. Beliefs, rituals, and repetitions of parents' and grandmothers' relationships with food appeared frequently.

On the one hand, occasionally, the three generations experienced pleasant moments together. Mealtimes with extended family had positive meanings. In these moments, there was less rigidity in the adolescent's act of eating.

Giselle: *It was on my birthday. We put the table outside. My grandmother made barbecue, my grandfather as well. Everybody was there and it was good! It was completely different, because we never eat like this*. (Anorexia nervosa).

Flora's mother: *There's noise, people talk, she plays with my niece* [at her grandmother's place]. (...) *She eats, she forgets the rigidity*. (Anorexia nervosa).

On the other hand, especially in the grandmothers' discourses, divergences about how the adolescent should be fed were evident. Grandmothers complained that their daughters did not transmit their recipes to their grandchildren:

Daniela's grandmother: *I taught her to make cinnamon tea in the morning and another one in the evening for her daughter. Each time my kids had were sick and vomiting when they were children, I would prepare cinnamon tea (...) I told her to do it, but she says she's always in a hurry and she can't do it*. (Bulimia Nervosa).

Less frequently, the ritual of praying was described in anorexia group. Meals were seen as beautiful moments for which they gave thanks by praying. Only one adolescent talked about the act of praying.

Flora: *I thank and I recite the Pater Noster*. (Anorexia nervosa).

Flora's mother: *Food, when you eat with pleasure, is something beautiful to see, which you eat with your eyes already, it requires a prayer for the soul*. (...) *I like to give thanks for the food, but it's more because of my family*. (Anorexia nervosa).

Another grandmother questioned the parents' educative attitudes. Some participants pointed out excessive paternal control not only over eating behavior, but also in more general personal development. During the interview, Valentina's grandmother frequently mentioned this to her son-in-law:

Valentina's grandmother: *You don't allow her to develop her own way of thinking because you control her too much!* (Anorexia Nervosa).

The act of eating was associated with sexuality. In the view of one grandmother, eating seemed to be important for being attractive:

Valentina's grandmother: *How are you going to find a boyfriend? The day he realizes there is nothing inside this little sponge, he will be disappointed with you. A man likes breasts and butt and she laughs!* (Anorexia nervosa)

The act of eating (and not eating) was associated with religious beliefs. For instance, one grandmother associated her granddaughter with the devil because of her anorexic symptoms. It affected on how the girl felt at the moment of eating.

Valentina: *It annoys me, I don't like it* (...) *My grandmother scolds me. She says I must eat, I must build up body mass, that I am far from God and I am close to the devil* [laughs]. (Anorexia nervosa)

Bulimic symptoms appeared as a factor of differentiating the adolescent from all the rest of the family.

Ester's grandmother: *My youngest daughter has little boys, they do not want to eat* [laughs] (...) *Asking them to eat is like declaring war as they refuse! Others are normal. Only* [she pronounces this word with an extended voice] *Ester is like that, she has this different way of eating*. (Bulimia nervosa).

Grandmothers complained that bulimic adolescents overate. They also prepared and gave large amounts of food to their grandchildren.

Ester's grandmother: [She said] *Grandma, is this plate for me? I said to her: Yes. She ate it all and got a bloated stomach! I saw it, she ate everything in the pan* [laughs]. *Then, after, she told me that she had a stomachache. I told her: that is because you ate too much!* (Bulimia nervosa).

## Discussion

The objective of this study was to understand the role of food in family relationships among Brazilian adolescents with anorexia and bulimia nervosa through photo elicitation. The superordinate themes of our study highlight the importance of control in nuclear and extended families on these teens' experiences with food.

Our results underline a specific association between eating disorders and mourning. Previous studies have explored this association, analyzing dimensions such as attachment and defense mechanisms in the context of eating disorders (43–45). It draws special attention to the relation of the origin and development of eating disorders to mourning, mainly, rather than to concerns about body image, which are very usual in this context. Food holds a very important place in a traumatic situation, in which the adolescents studied tried to create new bonds with their parents by taking control of their relationships.

In this study, eating behavior seemed to be underpinned by two paradoxical and simultaneous drives for the adolescents: trying both to control others and resisting other's control over them. It implies in a fight for autonomy and regulation of dependence toward parents, which is also found in previous studies with other groups of our research team, such as in adolescents with obesity (27, 28) and the study about the role of food in families with eating disorders or not (29). This struggle is marked by a loss of control by these teens and their parents. The difference is how the autonomy-dependency struggle takes place for the adolescents with BN and AN. In the BN group, this is strongly marked by parents' ambivalent attitudes, differently from the AN one. In this struggle, receiving some affection from parents appeared as a way which allowed adolescent girls with anorexia nervosa to eat, although it also implied an experience of suffering for these teens. At the same time, parents' acknowledgment of the suffering that adolescents with anorexia have while eating, through their expression of displeasure, does not prevent them from thinking about changing how they watch their children. Among our participants, watchfulness was more evident in the anorexia nervosa group than in the bulimia nervosa one.

One of the most important results of this study is the fathers' involvement: their control over the experiences with food of their daughters with anorexia. Historical studies have focused on mother-daughter relationships in the etiology of eating disorders and on the idea that the mothers of young people with anorexia do not respond to their children's psychological needs and messages (11, 46). The mothers are often described as overinvolved and enmeshed, and the fathers as cold and distant (11). A few previous studies have explored the paternal role within the context of eating disorders (47, 48). In one of these studies, patients reported that fathers were less caring and more overprotective, as well as less benevolent and more punitive (47).

Food seemed to be a vector of paternal intrusiveness, hampering patients' autonomy in eating in the group of anorexia nervosa. Fathers also expressed negative emotions about eating with their daughters and were especially concerned about the teens' aggressiveness toward them. In contrast, previous studies reported an important alexithymia among fathers of adolescent girls with eating disorders (49, 50).

Fathers' negative comments about the body shape of these girls with bulimia nervosa were associated with their negative eating experiences. Bulimic symptoms seemed to be a way of dealing with negative comments in a vicious circle. This finding is convergent with studies indicating that bulimic adolescents have experiences of vulnerability, shame, and rejection in their relationship with their fathers (51, 52). We also observed that they experienced these feelings with their mothers. These adolescents' experiences of eating seemed to be strongly accompanied by these feelings.

The strong parental control in the families dealing with anorexia nervosa led parents to make the family eat together as a means of surveillance. Family cohesion thus resulted more from obligation than from moments of pleasure and exchanges among family members. Poor communication and low frequency of acts of affection from parents toward their children were described. Family cohesion was, therefore, low, paradoxical, and conflictual. Thus, simultaneously an obligatory ritual brought the family together at mealtime and this ritual was a source of conflict between parents and adolescent girls, especially in the group of anorexia nervosa.

The low degree of cohesion observed in our study distinguishes our findings from previous data highlighting high cohesion in families dealing with anorexia (11, 53, 54). In the families we studied, conflicts around food were necessary to maintain a functioning family system in both groups. Food was an important means of reinforcing the impoverishment of family bonds. The lack of family identity through food brought experiences of sadness, mainly for parents and grandmothers, and anger for adolescent girls in the act of eating together. It contrasted with previous studies indicating that there was conflict avoidance in families with a member presenting anorexia nervosa (11, 29, 30, 55). Both adolescent girls and their parents recognize their conflicts, but it seemed they did not know how to manage them. It is important to develop strategies focused on how these families deal with their conflicts and change their dysfunctional patterns in order to favor an increase in the sense of family identity, without disregarding the individual identity.

In the bulimia nervosa group, we also observed there was no conflict avoidance, as seen in the study with French adolescent girls using the same methods (31), although there was a mechanism of avoidance of family members in some teens in our study. They did not confront the teenager for fear of the reactions that she may have in face of criticism. Different from previous findings (56), adolescents with BN perceived that they have conflicts within their families, as the AN group.

In most of the families in our study, we observed that their mealtimes were a space of confrontation for both groups. There was an emotional reactivity described by teens in this study, even though it varied between the two groups. Adolescent girls with bulimia had a strong emotional reactivity during family mealtimes and regarding questions not only focused on food, but they had more difficulties in expressing their emotions toward food and their families during the interviews. The emotional reactivity in anorexia did not appear in the other study with French teens using the same methods, in which all attempts at opposition focused on the issue of food (30). It also differs from studies showing an inhibited expression of negative emotions in anorexia (57, 58). The aspects of variability related to no conflict avoidance and emotional reactivity reinforce the cultural aspect of eating experiences of adolescent girls with eating disorders from different countries.

Teenagers' childhood experiences with their parents and memories of these parents about their own childhood and adolescence mealtimes are manifested in attitudes and behavior in their relationship with food. Both teens and parents have considered their memories as painful experiences, in which food seemed to be a means of suffering physical and, especially, psychological violence. As situations of mourning were observed in this study, these aspects of different violence experiences may seem as traumatic events which have an impact on how they construct their relationship with food. These findings have the same direction of those related to the association between trauma and weight status among adolescents in eating disorder treatments (59).

As a strength of our study, we observed remarkable disparities in the views of family members in three generations on how to eat and what rules and rituals to follow at mealtimes, bringing eating experiences marked by a feeling of discomfort among them. These results show a broader generational dimension of the conflicts in feeding patterns and family bonds. Experiences of eating seemed to be experiences of the intrusiveness of generational boundaries. Previous studies reported an association between care and communication by maternal grandmothers and eating disorders (60, 61). The grandmothers we interviewed exerted marked control over the patients, contributing to the pleasure or displeasure of these girls' relationships with food. The eating experiences are strongly tied to cultural concepts of body image which changes with generations. There is also a confrontation in the dyad grandmother-grandchild concerning viewpoints about what they consider as a healthy meal and about ideal body, which is different between these two generations. Less frequently, paradoxically, positive eating experience were described by parents and teens with extended families, especially in families of those with anorexia. These experiences meant less rigidity about rules and rituals around food and more flexibility about what and how to eat.

Previous studies with Brazilian families in general have highlighted that commensality is an important practice that appears in different settings and even in precarity contexts (62). In addition, Brazilian family mealtimes have changed. Aspects of contemporary lifestyles, such as individualization of meals and little time for food, coexist with valorization and maintenance of traditional meals (63). This study's results show an important conflict in which the parents' and grandparents' generations value commensality and traditional meals while adolescent girls try to keep their distance at family mealtimes and defy manners and the notion of hierarchy at the table, but this aspect varied for anorexia nervosa and bulimia nervosa. Eating together with family was more frequent in the anorexia group than those of bulimia one, marked by more individual meals. In addition, cultural factors such as religious rituals around Brazilian meals appeared less in this research, in contrast to a study, using the same methods, of Brazilian adolescents with obesity (28), in which the religious aspect seemed to be a vector for family cohesion and family mealtime organization, in contrast to the families of the adolescents interviewed for this study.

We observed an impoverishment of rituals and dietary rules at the level of the nuclear family, which led us to reflect on the impoverishment of the emotional ties experienced within the family and therefore to view the cohesion as fragile.

This study has limitations. Some themes may have been overrepresented by parents more than adolescents. This might have been associated with the girls' difficulties in expressing feelings and affective aspects. Another limitation is that only five grandmothers participated in this study, which restricted the understanding of their view in this study and the transgenerational aspects of eating. Some grandmothers were not available for interview. In addition, it was not possible to interview grandfathers: some adolescent girls did not have grandfather anymore; other grandfathers were not available for interview or didn't want to participate because they felt they were not able to speak about mealtimes. Some fathers also were not accessible to participate and one of them had died. We did not ask about adolescents' comorbidities in the interviews. Although our study did not aim at analyzing the eating experiences considering comorbidities, such as depression and anxiety, the interviews showed the need for further studies about their impact in family meals in the context of eating disorders. Subset analyses of each eating disorder may also be useful to better understand their specificities. Finally, the small sample allowed in depth analysis of the situations, but generalization must be done carefully.

Our study presents specifics about how nuclear and extended families are involved in the care of the adolescent Brazilian girls with eating disorders recruited for it and the conflicts they experience across three generations, which have a strong impact on these teens' relationships with food. This did not appear in previous studies by our research group. In addition, in this research, it was more evident that the interviewed families did not avoid conflict in their relationships in either group. Specifically, in the anorexia group, the main difference observed was that, in the study with French teens (30), seating at family mealtimes was fixed and changing it strongly discouraged. This was not observed in either of our study's groups.

Our original findings suggest that food potentiates conflicts between three generations in the families of teens with anorexia and bulimia in Brazil. Our data reinforce the need to develop therapeutic strategies in the treatment, focusing on the adolescent's autonomy in the family context and on the family identity related to food among three generations. It also implies a reorganization of boundaries among these generations in the treatment of eating disorders. It allows a space for autonomy and identity at the individual level, without disregarding family identity through food. It is important to have parents' participation in monitoring their daughters' meals for their weight restoration in the case of AN and for disrupting binge eating, purging, and restrictive dieting in the case of BN. As weight gain and bulimic episodes are controlled, parents must be encouraged to promote a healthier and more pleasurable individual relationship between their daughters and food. This can enable teens find pleasure in the act of eating not only in their family relationships, but also, in their relationships with their peers. Furthermore, at the individual level, it is important to develop interventions focusing on emotional reactivity. Future studies that can analyze paternal and maternal grandmothers' and mothers' relationships with food in their families are needed. It is also important to develop research evaluating different cultural contexts in the investigation of how experiences with food are lived and transmitted among three generations.

## Data Availability Statement

The raw data supporting the conclusions of this article will be made available by the authors, without undue reservation.

## Ethics Statement

The study was approved under number 607.183 by the Ethics Review Committee of Universidade Federal do Ceará in Fortaleza, Brazil, at 15th October 2013, according to Resolution 466 of the National Council, and written informed consent was obtained from all participants and parents where necessary (in the case of minors).

## Author Contributions

JL, MM, and JB-M: study design. JR, ME, LB, and JL: drafting manuscript. JR: data analysis and study conduct. All authors: approving final version of manuscript, revising manuscript content, and data interpretation.

## Conflict of Interest

The authors declare that the research was conducted in the absence of any commercial or financial relationships that could be construed as a potential conflict of interest.
